# Uveitic glaucoma in children: a systematic review on surgical outcomes

**DOI:** 10.1186/s12348-022-00313-2

**Published:** 2022-11-07

**Authors:** Charlotte L. L. I. van Meerwijk, Nomdo M. Jansonius, Leonoor I. Los

**Affiliations:** Department of Ophthalmology, University Medical Center Groningen, University of Groningen, P.O. Box 30.001, 9700 RB Groningen, The Netherlands

**Keywords:** Pediatric uveitis, Childhood, Glaucoma, Surgery, Surgical outcomes

## Abstract

**Purpose:**

To compare the outcomes and complications of different surgical interventions for secondary glaucoma in pediatric uveitis.

**Methods:**

Systematic review following the PRISMA standards. Main inclusion criteria were surgery for secondary glaucoma in pediatric uveitis at a mean age of 16 years or below, a mean follow-up period of at least 1 year after surgery, and at least 10 eyes per surgical intervention per study. We used the GRADE approach to assess study quality. Primary outcomes were intraocular pressure (IOP) and number of IOP lowering medications before and after surgery. Secondary outcomes were success rate and complications.

**Results:**

Fourteen studies fulfilled the inclusion criteria, in which one (*n* = 11) or more (*n* = 3) surgical interventions were described, comprising in total six different procedures. According to the GRADE criteria, the quality of the studies was low to very low, in particular because of the small size and the applied study designs. All surgical interventions provided a significant decrease in IOP and number of IOP lowering medications. The success rates during follow-up varied widely, with the lowest rates of success after cyclophotocoagulation. The most frequently reported complications were ocular hypertension, hypotony, and hyphema, with an indication for a reoperation in more than one-third of the cases. Permanent vision loss was infrequently seen and was attributed to prolonged hypotony.

**Conclusions:**

The described surgical interventions are able to prevent blindness by lowering a medically uncontrolled IOP to an acceptable level. Therefore, there is a crucial role for surgical intervention in these children. Based on the present studies, no preferences can be made. Given the reported complications, more research with larger sample sizes and direct comparisons is needed to determine the most successful glaucoma treatment in children with uveitis.

**Supplementary Information:**

The online version contains supplementary material available at 10.1186/s12348-022-00313-2.

## Background

The incidence of pediatric uveitis is 4.3–6 per 100,000 person-years [1, 2], which is 5–10% of the uveitis population [3]. Pediatric uveitis is a challenge to treat, because of the often delayed diagnosis due to asymptomatic presentation, ocular evaluation difficulties, treatment difficulties caused by age and side effects, and the risk of serious ocular complications. Secondary glaucoma is one of the most serious and potentially blinding complications in pediatric uveitis [4, 5]. Based on a retrospective analysis in a tertiary care referral center, secondary glaucoma developed in 26% of the children with uveitis [6].

Uveitic glaucoma develops as a result of increased resistance to the aqueous humor outflow by several mechanisms, including mechanical obstruction due to entrapment of inflammatory cells and debris in the trabecular meshwork, swelling of the meshwork itself, and secondary scarring or collapse of the trabecular meshwork and/or Schlemm’s canal. Also, treatment with corticosteroids may increase outflow resistance by modifying the trabecular meshwork. This type of glaucoma is often hard to control; 30–61% of patients with this type of secondary glaucoma needed a surgical intervention in addition to pharmacological treatment [6–8]. Hence, a detailed knowledge of the advantages and disadvantages of the various surgical options is pivotal.

Different surgical interventions are described in pediatric uveitis glaucoma. The surgical interventions can be subdivided into four categories: angle surgery, fistulizing surgery, glaucoma drainage implants (GDI), and cyclodestructive/cyclophotocoagulation (CPC) procedures. The goal in angle surgery is to facilitate entrance of aqueous humor into Schlemm’s canal. Trabeculodialysis is the first chamber angle surgery technique described, with treatment performed at the level of the Schwalbe’s line. During goniotomy, the anterior trabecular meshwork is incised just below Schwalbe’s line by using a knife/needle under direct visualization of the chamber angle with a gonioscopy lens. Goniotomy is usually performed initially over 4 to 5 clock hours and can be repeated (extended to more clock hours) [9]. During trabeculotomy, Schlemm’s canal is entered with a probe via an external approach. Then the probe is rotated into the anterior chamber, cleaving the trabecular meshwork. The aim of fistulizing procedures is to create a pathway for external drainage of aqueous humor from the anterior chamber to the subconjunctival space. During a trabeculectomy (TE), a peripheral iridectomy, a scleral ostium, and a scleral flap are created, after which the scleral flap is sutured back to its original position. After surgery, the aqueous humor flows into the subconjunctival space, leading to an elevation of the conjunctiva (filtering bleb). Over time a local fibrous capsule is formed. A GDI creates a pathway for external drainage via a device. All GDIs are based on a silicon tube placed in the anterior chamber, in the posterior chamber (between iris and lens), or in the vitrectomized posterior segment via the pars plana, in order to shunt aqueous humor to the subconjunctival space, where the tube is connected to a plate positioned at the equator region of the eyeball.

A fibrous capsule forms around this plate. The size and material of the plate vary between the different devices. The first drainage system utilized was the Molteno non-valved design in 1970. Currently, the most utilized devices are the non-valved Molteno and Baerveldt and the valved Ahmed device [10]. All implants are available in a smaller version, targeting eyes with increased hypotony risk. The CPC procedure is designed to reduce aqueous humor production by destruction of the ciliary body. The treatment can be performed with an endoscopic or a transscleral technique. In the endoscopic CPC procedure, a probe is introduced into the anterior chamber, after which laser energy is directly applied to the ciliary body until shrinkage and whitening occur [11]. During the transscleral CPC procedure, the laser probe is placed externally against the sclera and laser energy is applied to the area of the ciliary body. Infrared light is absorbed by the pigmented epithelial cells of the ciliary body, causing destruction of ciliary body epithelium by coagulation necrosis [12].

Surgery is a challenge given the young age of the patients and the underlying uveitis. The young age itself is a critical component of the management, due to a different response to surgery and medication, the need for general anesthesia, and the often challenging cooperation during pre- and post-op evaluations. In addition, it is essential to control the underlying uveitis as optimally as possible, whereas active uveitis may lead to early failure of the operation [13]. The damaged trabecular meshwork together with ciliary body dysfunction, caused by chronic uveitis and/or previous IOP-lowering interventions, makes the balance between ocular hypertension and hypotony even more fragile.

To gain a better understanding of the currently available surgical strategies and their outcomes, we performed a systematic review of postoperative outcomes and complications in different surgical strategies in pediatric uveitis patients.

## Methods

We performed a systematic review of the literature. Inclusion criteria for data abstraction were as follows: (1) the study reported outcomes after a surgical intervention for secondary glaucoma in patients with pediatric uveitis (mean age 16 years or below at uveitis diagnosis), (2) the study reported data on intraocular pressure (IOP), IOP lowering medication, and/or complication rates, (3) the study reported original research, (4) the study had a mean follow-up period of at least 1 year after surgery, and (5) at least 10 eyes per surgical intervention were included. One researcher performed the literature search in PubMed and Embase with the search string "Child"[Mesh] AND "Uveitis"[Mesh] AND "Glaucoma"[Mesh]”. Additional search was conducted through the reference lists of the identified articles. The search was performed in January, 2022. The inclusion of the studies from our search was based on the ‘Preferred Reporting Items for Systematic Reviews and Meta-Analyses’ (PRISMA)-procedure [14].

Primary outcome measures were IOP and the number of IOP lowering medications before surgery, 1 year (9 to 15 months) after surgery, 2 years (18–30 months) after surgery, and 5 years (48–72 months) after surgery. Secondary outcome measures were surgical success rates at the same time points and complications during follow-up.

Quality of evidence-grading was based on the GRADE approach [15–18] which consists of four levels: high, moderate, low, or very low quality. Major aspects that influence the level of quality are the risk of bias, inconsistency, indirectness, imprecision, publication bias, and study design.

## Results

**Fig. 1:**
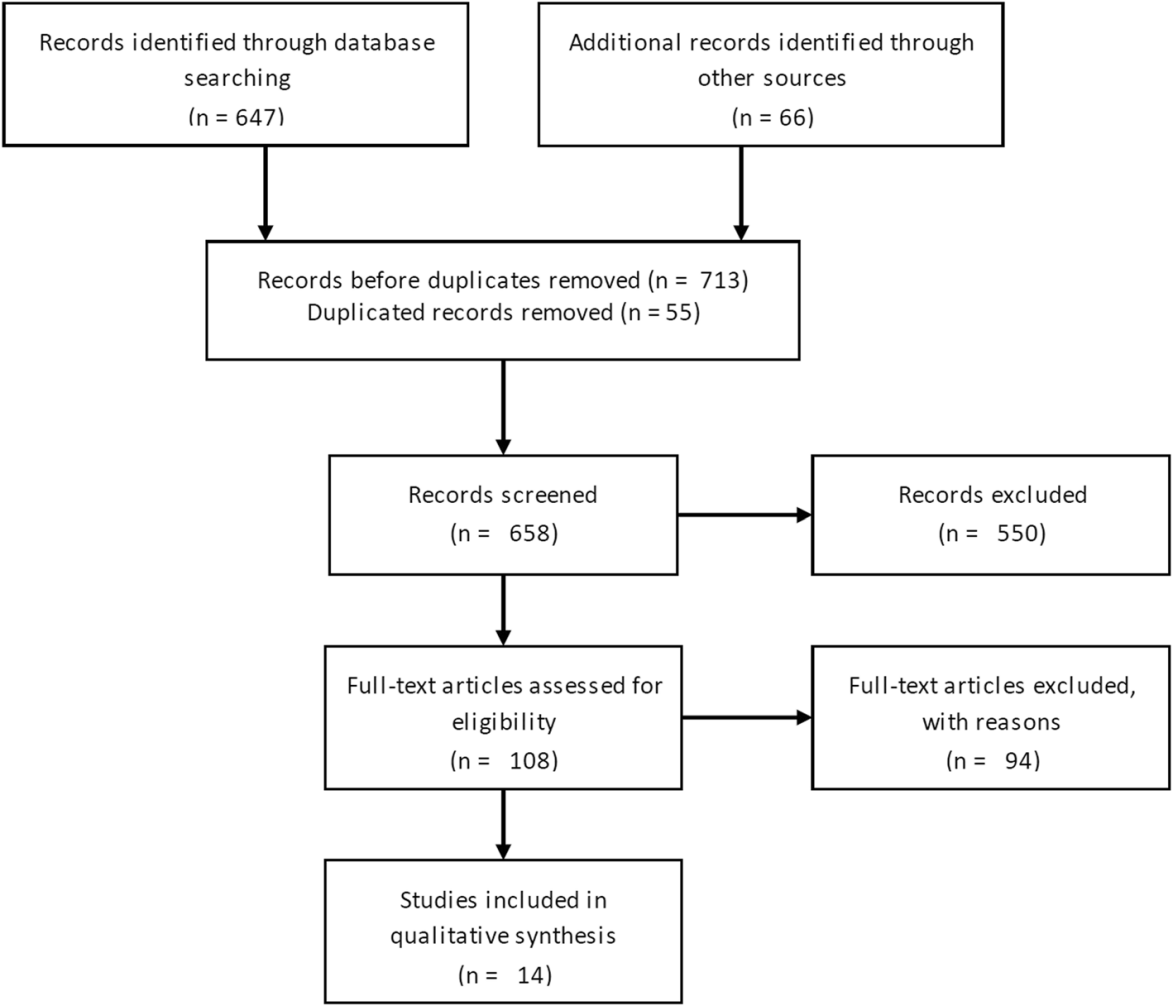
Literature search, PRISMA flow diagram

The literature search resulted in 713 potentially relevant articles (Fig. 1). Fourteen studies fulfilled the inclusion criteria, in which one (*n* = 11) or more (*n* = 3) surgical interventions were described, comprising in total six different procedures. Of the articles on multiple surgical procedures, one publication [19] fulfilled the inclusion criteria in two different surgical interventions. One research group published two different articles, with different research questions on the same surgical intervention. Because the two articles complemented each other in content and were published at short intervals, we combined the results of these two publications [20, 21]. None of the included publications used a randomized controlled or a solid comparative study design.

The lack of statistical comparison leads to an inability to calculate the effects of different surgical interventions, and therefore no quality assessment can be carried out. According to the ‘Grading of Recommendations Assessment’ (GRADE) approach, all studies have a very low quality grading.

Table 1 shows all included studies, divided into the previously mentioned four categories of surgical interventions. All but one included studies evaluated a defined study group consisting of patients with pediatric uveitis who developed secondary glaucoma; only Chen et al. [22] evaluated a general pediatric glaucoma patient group, and included a subgroup analysis of pediatric uveitis patients. The included studies used diverse definitions of surgical success (Table 1). Some studies distinguish between success and qualified success, with qualified success using a less strict definition of success (with respect to the IOP and/or additional IOP-lowering medication). The number of previous interventions performed before the interventions of interest varied. In most cases, angle surgery and a CPC procedure were the first surgical interventions performed (Table 1). In contrast, TE and GDI procedures often followed previous interventions.

**Table 1:** Studies on surgical interventions in pediatric uveitic glaucoma – success rates

						**Criteria for success (C = complete / Q = qualified)**				**Success rate**		
**Author, Year**	**Type of surgery**	**Previous interventions**	**No. of Eyes**	**No. of Patients**	**Age at Surgery (yrs)**	**IOP (mmHg)**	**Med**^a^	**No. of procedures**^b^	**Other**	**1 yr**	**2 yrs**	** ≥ 5 yrs**
Kanski and McAllister,’85 [23]	Trabeculo- dialysis	43% CPC^b^,37% filtration surgery	30	23	16 (4–36)^c^	< 22	C: 0 Q: no limitation	1			C: 17% Q: 60%	
Freedman et al.,’02 [9]	Goniotomy	no	16	12	15 (6.5–30)^c^	< 22, > 5	no limitation	1–2	no adverse events	1x: 72%^d^ 2x: 79%	1x: 54%^d^ 2x: 75%	1x:54%^d^ 2x:75%
Ho et al.,’04 [20, 21]	Goniotomy	few cases had a TE before	40	31	10.3 ± 4.7^e^	< 22, > 5	no limitation	1–3		> 1x:92%	1x: 61%^d^ > 1x: 92%	1: 47%^d^ > 1x:81%
Bohnsack and Freedman,’13 [24]	Goniotomy	no	31	31	10.9 ± 4.4^e^	< 22, > 5	no limitation	1–2	no adverse events	1x: 76%^d^ 2x: 83%	1x: 57%^d^ 2x: 79%	1x: 48%^d^ 2x: 69%
Wang et al.,’16 [25]	Trabeculo-tomy	18% goniotomy 7% Ahmed implant	28	22	9.7 ± 3.6^e^	< 22, > 5	no limitation	1–2		1x: 81%^d^ 2x: 86%	1x: 69%^d^ 2x: 77%	
Heinz et al.,’11 [26]	TE^f^	88% CPC^b^	16	16	12.7 ± 3.8	< 22, > 5	C: 0 Q: no limitation	1			C: 88% Q: 94%	
Wiese et al., ‘14 + ’16 [19, 27]	TE^f^	61% CPC^b^	21	17	13.8 ± 4^e^	< 15, > 5	C: 0 Q: no limitation	1		C: 55% Q: 70%		C: 38% Q: 71%
Leinonen et al.,’15 [28]	TE^f^	24% CPC^b^, 7% viscocanalostomy	15^d^ 14^f^	15^d^ 14^f^	11.8 (6.3–20.4)^g,c^ 11.0 (3.1–16.8)^c,h^	< 22, > 5	0	1		73%^g^ 57%^h^		73%^g^ 16%^h^
Gautam et al.,’18 [6]	TE^f^	no	13	9	≤ 16	< 22, > 5	C: 0 Q: no limitation	1			C: 54%^i Q: 23%	
Valimaki et al., ‘97 [29]	Molteno shunt	52% TE^h^, 22% CPC^b^	27	19	15.3 ± 8.7 ^e^	< 22, > 5	No more as used pre-op^j^	1	no loss of light perception		95%	89%
Chen et al.,’15 [22]	Ahmed implant		24		9.0 ± 3.8 ^e^	< 22, > 5 + 20% IOP reduction	no limitation	1 + revision of GDI accepted	no adverse events			75%
Wiese et al.,’16 [19]	Ahmed implant	54% TE^h^, 45% CPC^b^, 27% goniotomy	11	11	17.8 ± 6.1^e^	< 15, > 5	0	1				
Eksioglu et al., ‘17 [30]	Ahmed implant	25% TE^h^	16	11	14.2 ± 3.3^e^	< 22, > 5	C: 0 Q: no limitation	1 + small revisions accepted		C: 56%		C: 38% Q: 44%
Heinz et al., ‘06 [31]	CPC procedure	no	19	12	10.9 ± 3.4^e^	< 22	C: 0 Q: no limitation	1–3		C: 0% Q: 32%		

^a^Number of IOP lowering medications that is accepted

^b^Cyclophotocoagulation

^c^Based on mean (range)

^d^After 1 or 2 procedures, based on Kaplan–Meier survival analysis

^e^Based on mean ± SD

^f^Trabeculectomy

^g^Group using Tumor Necrosis Factor Inhibitors

^h^Control group without the use of TNFI

^i^After a mean follow-up of 2.3 ± 1.6 years

^j^Before operation

### Primary outcomes

**Fig. 2:**
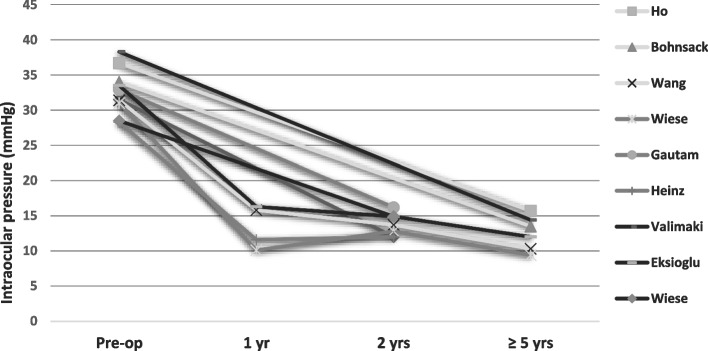
Intraocular pressure during follow-up

**Fig. 3:**
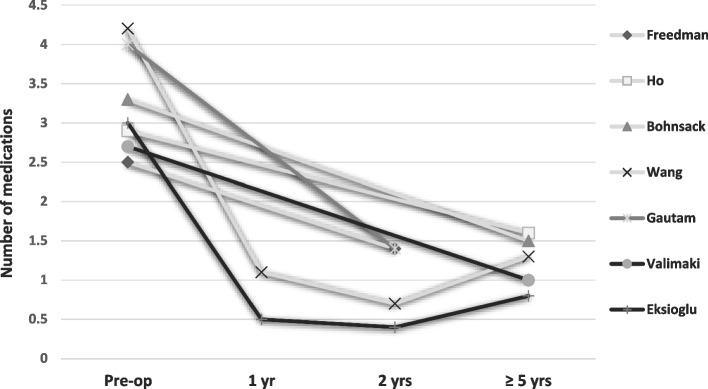
Intraocular pressure- lowering medication during follow-up

Figures 2 and 3 give the IOP and number of IOP-lowering medications used during follow-up in the included studies. More details are listed in supplementary Table S1.

Overall, after all surgical interventions, a significant IOP reduction was achieved, together with a significant reduction in the need of IOP-lowering medication. In most cases, a small number of IOP-lowering medications was still necessary. The study based on the CPC procedure only published the IOP and IOP-lowering medication before the next CPC procedure (mean of 2.25 procedures per eye). The authors measured a significant reduction in IOP and IOP lowering medications after a CPC procedure [31].

### Success rates

Success definitions and thereby the success rates in the included articles were quite diverse (Table 1), which makes it impossible to perform a statistical comparison.

In the angle surgery studies, the success rate at 1 year after surgical intervention was 76–90%, at 2 years 17–77%, and at ≥ 5 years 48–81%. A higher success rate was seen after more than one goniotomy procedure [24]. Conflicting results were reported concerning the effect of the following variables on success rate: preoperative IOP of > 21 mmHg [worse outcome: Kanski and McAllister [23]; no effect: Freedman et al. [9], presence of peripheral anterior synechiae [worse outcome: Ho et al. [21], Bohnsack and Freedman [24], Wang et al. [25]; no effect: Kanski and McAllister [23], Lens status: aphakia [worse outcome: Ho et al. [21]; no effect: Bohnsack and Freedman [24], Kanski and McAllister [23], Freedman et al. [9], patients younger than 10 years of age at the time of surgery [better outcome: Ho et al. [21]; no effect: Kanski and McAllister [23], Freedman et al. [9], longer duration between the onset of uveitis and the surgery [worse outcome: Ho and Walton [20] (without a persistent significant result after stratifying by lens removal (yes/no)); no effect: Freedman et al. [9], longer duration between the glaucoma diagnosis and the surgery [worse outcome: Ho and Walton [20]; no effect: Freedman et al. [9]. In one study, a worse IOP control was seen in those with prior surgery [21] and in idiopathic anterior uveitis [25]. In addition, the following variables were examined, without finding an association with the success rate: race [9], age of onset of uveitis [20], preoperative posterior synechiae [9] and the duration of preoperative uveitis control [9].

In the fistulizing procedure studies, the success rate at 1 year after surgical intervention was 55–73%, at 2 years 39–88%, and at ≥ 5 years 16–73%. In a sub-analysis, Leinonen et al. [28] described a significantly better outcome with the use of Tumor Necrosis Factor (TNF) inhibition. The use of TNF inhibition was not significantly associated with postoperative hypotony. No association was observed with any previous surgery on the operated eye [27, 28]. Wiese [27] described no differences in outcomes in children with aphakic eyes as a subgroup, compared to the total study group (*n* = 11 phakic/ *n* = 3 pseudophakic/ *n* = 7 aphakic).

In the GDI procedure studies, the success rate at 1 year after surgical intervention was 56% [30], at 2 years 95% [29], and at ≥ 5 years 38–89% [22, 29, 30]. No additional information was published about possible risk factors for failure.

In the CPC study, the success and qualified success rates at 1 year were 0 and 32%, respectively. The lens status, uveitic complications, other complications, or treatment with immunosuppression did not influence the outcomes. When compared to the other surgical techniques as described above, the lowest success rates after one year were in the CPC study group.

### Complications and reoperations

Table 2 lists the most common complications and the number of reoperations. Hypotony was the most common complication. The highest incidences of postoperative hypotony were reported after fistulizing procedures (8–71%). In most cases, hypotony was temporary and did not result in a permanent decrease in visual acuity. A reopertion for hypotony was only described by Wiese et al. [27] in 5/21 eyes after TE and by Eksioglu et al. [30] in 1/16 eyes after a GDI during early follow-up. Frequent reoperations were needed during follow-up in all surgical categories, either because of the IOP management (most often hypertension) or because of complications.

**Table 2:** Post-operative complications

Complication		Intervention type			
		**Angle surgery**	**Fistulizing procedures**	**GDI procedures**	**CPC procedures**
Hyphema		56–80%^a^	7–14%	6–11%	
Hypotony			8–72%	13–18%	
	*Shallow anterior chamber*		14–40%	6–11%	
	*Choroidal detachment*	4–24%	0–50%	4%	
	*Papilledema*		25%		
Vitreous prolapse			13%		
Infection				6%	
Cataract progression		6–21%	31–48%	11–50%	11%
Bleb: Tenon cysts			6%		
Tube	*Tube block by iris or vitreous*			6–11%	
	*Cornea-tube touch*			6–11%	
	*Tube exposure*			25%	
Reoperation		29–48%	43–62%	38%	37%

^a^Mostly transient hyphema after angle surgery, only 6% had a total hyphema complicated by increased IOP requiring an anterior chamber washout or another surgical intervention (Ho et al.). *GDI* Glaucoma drainage implants, *CPC* Cyclodestructive/cyclophotocoagulation procedure

## Discussion

All described surgical interventions lead to a decrease in IOP and a decrease in the number of IOP lowering medications. Overall, the risk of complications and re-interventions is high. Because of small study sizes and differences in study design and definitions, no good comparisons were possible and no clear preference for one method can be given. Taking these limitations into account, CPC seems the least effective technique.

In the included studies, hypotony is the most common complication. The highest incidences of postoperative hypotony were reported after fistulizing procedures (8–71%), the lowest incidence of zero percent was reported after a CPC procedure. To prevent hypotony after GDI surgery, most often a valved Ahmed implant was used (Table 1) [10]. Of all cases of hypotony described, only two studies reported the need for an additional intervention. Hypotony is mostly temporary and without permanent visual loss, and may be acceptable.

Although re-operations were seen after all interventions, higher re-intervention rates were described after a CPC procedure and after fistulizing procedures, but no reliable statistical comparisons could be made. The large re-intervention rates after a CPC-procedure were mainly based on high failure rates, with persistent high IOP after one treatment. The large re-intervention rates after fistulizing procedures were partly due to the fragile balance between too little and too much aqueous humor drainage, which makes the management after surgery challenging and often requires minor adjustments to the filtering bleb. The frequent need of additional procedures should be taken into account, especially in young patients who often require hospitalization and general anesthesia for minor and major surgical procedures. In addition, changes in therapy, ophthalmic or systemic discomfort, and frequent visits to the hospital can have a high impact on the quality of life.

The control of uveitis and the degree of active inflammation is a potential aspect influencing outcomes after glaucoma surgery [13]. Three out of five studies on angle surgery describe a correlation of the outcomes with the presence of peripheral anterior synechiae, which is a sign of previous uncontrolled inflammation. After filtering surgery, Leinonen et al. [28] described a significantly higher success rate in the TNFI group, which supports the theory that reduction of fibrosis postoperatively due to a more effective control of systemic inflammation could increase the IOP reduction and lower the risk of failure. The level of inflammation and its suppression by disease-modifying anti-rheumatic drugs (DMARDS) could be an important factor, related to the complications and ciliary body damage present before surgery [32] and for the survival of filtering blebs and the occurrence of fibrosis after filtering surgery [33–35].

More research is needed to establish the precise impact of DMARDS on the development of secondary glaucoma, perioperatively and on the long-term success. Apart from the medication, the type of surgical intervention could also influence the amount of inflammation after surgery. Wiese et al. [19] made a comparison of the degree of flare and anterior chamber cells after a TE and an Ahmed drainage implant, with only a significant reduction measured after a TE.

Another potential aspect influencing outcomes after glaucoma surgery is lens status. Heinz et al. [26] advise against a TE in case of aphakia, due to a risk of a vitreous prolapse resulting in failure. From our included studies, seven studies have investigated lens status as a potential risk factor for early failure, of which only one showed significant differences in outcome in favor of phakic eyes during a goniotomy procedure [21]. However, in more recent studies, cases have already been selected partly on the basis of their lens status, following the early mentioned advice, so a reliable comparison can no longer be made. Cataract formation is often described after surgery. Given the multifactorial nature of cataract development in children with uveitis, a direct correlation with different types of glaucoma surgery is difficult to establish. However, the development of cataract can influence the follow-up. In fact, a higher TE failure rate after cataract surgery has been described in adults [36, 37]. The cataract progression rate of 31–48% in our included studies on TE and therefore the need for cataract surgery after a TE procedure could possibly influence the risk of failure.

A rare, but potentially blinding complication is the risk of infection. In the reviewed studies, postoperative endophthalmitis was described after 28 months of surgery in only one eye [30]. Late endophthalmitis is primarily described after TE, which carries a lifelong risk of filtering bleb leaks or infections, especially when anti-scarring agents have been used. In non-uveitis populations, patients who underwent a TE at childhood had a higher risk of endophthalmitis, with an increasing risk in those wearing contact lenses [38]. Twenty-one percent of the Dutch citizens wear contact lenses with a peak between 16 and 40 years of age [39]. Therefore, this aspect must be taken into account. In GDI, tube exposure is correlated with endophthalmitis, and this is significantly more often present in patients with uveitis [40]. A specific complication after GDI is an ocular motility disturbance, for which 3% of the patients underwent strabismus surgery during follow-up [22]. Our included studies do not reflect these complications, probably because of the small study groups and relatively short follow-up.

Based on our results, no evidence based advice of preference can be formulated between angle, fistulizing and GDI surgery. TE and GDI surgery are the most commonly used techniques in contemporary practice in children with uveitic glaucoma [41–43]. There is considerable experience with these techniques in both adults and children. Also, a stepwise approach may be considered, starting with a less invasive procedure such as angle surgery, and—if necessary—performing a more invasive procedure when the child is older.

All studies had a very low quality of evidence; the study groups were small and conclusions were based on retrospective analyses. This implies that the risks of bias, inconsistency, and imprecision are considerable in all study groups. A meta-analysis cannot be performed because of small study groups and differences in definitions and outcome measures. To improve the quality of the evidence, more uniform study designs are needed with a comparable definition of success, time points of measurement, risk factor analysis and complications and re-intervention reports. Studies with a prospective design, larger study groups, comparison studies and/or randomized controlled trials would give more reliable evidence about different techniques that can be used in secondary glaucoma in children with chronic uveitis.

## Conclusions

In secondary glaucoma in pediatric uveitis, life-long adequate IOP regulation is essential. Adequate IOP regulation is difficult in the vulnerable eyes of children with chronic uveitis, leading to a very fragile balance between ocular hypertension and hypotony. Whereas the success rates of glaucoma surgery, the need for revision and the complication rates are far from optimal, the surgical interventions described in our review are able to prevent blindness by lowering the IOP to an acceptable level. Therefore, there is a crucial role for surgical intervention in these children. The wide range of outcomes reflects the huge diversity of the surgical techniques and study designs. It can be carefully stated that the CPC procedure has the lowest success rate compared to the other types of surgical interventions, and therefore is not preferred for secondary glaucoma in pediatric uveitis. However, more research is necessary to differentiate between the outcomes of the other surgical interventions.

## Supplementary Information

**Additional file 1:** **Table S1.** Studies on surgical interventions in pediatric uveitic glaucoma – primary outcomes.

## Data Availability

Data sharing is not applicable to this article as no datasets were generated or analysed during the current study.

## Abbreviations

CPC: Cyclodestructive/cyclophotocoagulation
DMARDS: Disease-modifying anti-rheumatic drugs
GDI: Glaucoma drainage implants
GRADE: The grading of recommendations Assessment, Development and Evaluation
IOP: Intraocular pressure
PRISMA: The preferred reporting items for systematic reviews and meta-analyses
TE: Trabeculectomy
TNFI: Tumor necrosis factor inhibition
