# Supplementary material for: Uveitic glaucoma in children: a systematic review on surgical outcomes
Source: J Ophthalmic Inflamm Infect. 2022 Nov 7;12:35. doi: 10.1186/s12348-022-00313-2 (PMC9640517; doi:10.1186/s12348-022-00313-2)
Supplement: Supplementary file 1 — Additional file 1: Table S1. Studies on surgical interventions in pediatric uveitic glaucoma – primary outcomes. [file 12348_2022_313_MOESM1_ESM.docx]

| **Table S1 Studies on surgical interventions in pediatric uveitic glaucoma – primary outcomes** | | | | | | | | |
| --- | --- | --- | --- | --- | --- | --- | --- | --- |
| **Author, Year** | **IOP pre-op^a,b^** | **IOP 1 yr post-op^a,b^** | **IOP 2 yrs post-op^a,b^** | **IOP ≥ 5 yrs post-op^a,b^** | **Med^c^ pre-op^a^** | **Med 1 yr post-op^a^** | **Med 2 yrs post-op^a^** | **Med ≥ 5 yrs post-op^a^** |
| **Angle surgery** | | | | | | | | |
| Kanski and McAllister, ‘85 | 17% < 21mmHg  83% > 21 mmHg |  | 60% < 21 mmHg |  | 17% drops only^d^  83% drops + systemic |  | 83% with med |  |
| Freedman et al, ‘02 | 31.5±4.3 |  | 12±2.5^e^ |  | 2.5±1.1 |  | 1.4±1.1^e^ |  |
| Ho et al, ‘04 | 36.7±6.4 |  |  | 15.7±3.1^f^ | 2.9±1.1 |  |  | 1.6±1.1^f^ |
| Bohnsack and Freedman, ‘13 | 34.0±7.8 |  |  | 13.5±4.5^g^ | 3.3±1.1 |  |  | 1.5±1.4 |
| Wang et al, ‘16 | 31.4±7.6 | 15.8±6.0 | 13.7±3.3 | 10.3±4.5 | 4.2±1.1 | 1.1±1.6 | 0.7±1.2 | 1.3±2.3 |
| **Fistulizing procedures** | | | | | | | | |
| Heinz et al, ’11 | 28.3±5.7 | 11.6±4.7 | ±12^h^ |  | 3.3 |  |  |  |
| Wiese et al, ‘14 + ‘16 | 31±6.7 | 10.1±4.7 | 12.8±6.1 | 9.4±.5.0 |  |  |  |  |
| Leinonen et al, ‘15 | 35 (23-45)^i,j^  33 (21-47)^i,k^ | 73% < 21^j^  64% < 21^k^ |  | 47% < 21^j^  14% < 21^k^ |  |  |  |  |
| Gautam et al, ‘18 | 32.9 |  | 16.2^g^ |  | 4 |  | 1.2±1.2^g^ |  |
| **GDI implantation** | | | | | | | | |
| Valimaki et al, ‘97 | 38.3±5.6 |  |  | 14.4±4.3^d^ | 2.7±0.9 |  |  | 1.0±1.1^d^ |
| Chen et al, ‘15 |  |  |  |  |  |  |  |  |
| Wiese et al, ‘16 | 28.5±5 |  | 14.9±6.6 |  |  |  |  |  |
| Eksioglu et al, ‘17 | 33.5±7.3 | 16.3±2.8 | 14.9±2.4 | 12±3.7 | 3±0.0 | 0.5±0.8 | 0.4±0.9^c^ | 0.8±1.3^l^ |
| **CPC procedures** | | | | | | | | |
| Heinz et al, ‘06 | 30.2±5.5 |  |  |  |  |  |  |  |
| ^a^ Pre of postoperative data, based on mean±SD, ^b^IOP in mmHg, ^c^Number of IOP lowering medications used, ^d^ Only topical therapy with timolol and carbonic anhydrase inhibitors as systemic therapy have been described,  ^e^After a mean follow-up of 32 (6-84) months, based on successful eyes, ^f^After a mean follow-up of 98.9±87.8 months, based on successful eyes with IOP lowering medication, ^g^After a mean follow-up of 2.3±1.6 years, ^h^Based on figure 1 in the article, no concrete number is given, based in patients without failure, ^i^Based on median(range), ^j^Group using Tumor Necrosis Factor Inhibitors (TNFI), ^k^Control group without the use of TNFI,  ^l^After 40 (6-116) months of follow-up | | | | | | | | |
